# Informing the public health response to COVID-19: a systematic review of risk factors for disease, severity, and mortality

**DOI:** 10.1186/s12879-021-05992-1

**Published:** 2021-04-12

**Authors:** M. Flook, C. Jackson, E. Vasileiou, C. R. Simpson, M. D. Muckian, U. Agrawal, C. McCowan, Y. Jia, J. L. K. Murray, L. D. Ritchie, C. Robertson, S. J. Stock, X. Wang, M. E. J. Woolhouse, A. Sheikh, H. R. Stagg

**Affiliations:** 1 Usher Institute, University of Edinburgh, 30 West Richmond Street, Edinburgh, EH8 9DX UK; 2 Medical Research Council Clinical Trials Unit, University College London, London, UK; 3 School of Health, Wellington Faculty of Health, Victoria University of Wellington, Wellington, New Zealand; 4 School of Medicine, University of St. Andrews, St. Andrews, UK; 5 Freelance consultant, Beijing, People’s Republic of China; 6 National Health Service Fife, Kirkcaldy, UK; 7 Public Health Scotland, Glasgow, UK; 8 School of Medicine and Dentistry, University of Aberdeen, Aberdeen, UK; 9 Department of Mathematics and Statistics, University of Strathclyde, Glasgow, UK; 10 School of Biological Sciences, University of Edinburgh, Edinburgh, UK

**Keywords:** Coronavirus, COVID-19, Systematic review, Review, Risk factors, Morbidity, Mortality

## Abstract

**Background:**

Severe Acute Respiratory Syndrome coronavirus-2 (SARS-CoV-2) has challenged public health agencies globally. In order to effectively target government responses, it is critical to identify the individuals most at risk of coronavirus disease-19 (COVID-19), developing severe clinical signs, and mortality. We undertook a systematic review of the literature to present the current status of scientific knowledge in these areas and describe the need for unified global approaches, moving forwards, as well as lessons learnt for future pandemics.

**Methods:**

Medline, Embase and Global Health were searched to the end of April 2020, as well as the Web of Science. Search terms were specific to the SARS-CoV-2 virus and COVID-19. Comparative studies of risk factors from any setting, population group and in any language were included. Titles, abstracts and full texts were screened by two reviewers and extracted in duplicate into a standardised form. Data were extracted on risk factors for COVID-19 disease, severe disease, or death and were narratively and descriptively synthesised.

**Results:**

One thousand two hundred and thirty-eight papers were identified post-deduplication. Thirty-three met our inclusion criteria, of which 26 were from China. Six assessed the risk of contracting the disease, 20 the risk of having severe disease and ten the risk of dying. Age, gender and co-morbidities were commonly assessed as risk factors. The weight of evidence showed increasing age to be associated with severe disease and mortality, and general comorbidities with mortality. Only seven studies presented multivariable analyses and power was generally limited. A wide range of definitions were used for disease severity.

**Conclusions:**

The volume of literature generated in the short time since the appearance of SARS-CoV-2 has been considerable. Many studies have sought to document the risk factors for COVID-19 disease, disease severity and mortality; age was the only risk factor based on robust studies and with a consistent body of evidence. Mechanistic studies are required to understand why age is such an important risk factor. At the start of pandemics, large, standardised, studies that use multivariable analyses are urgently needed so that the populations most at risk can be rapidly protected.

**Registration:**

This review was registered on PROSPERO as CRD42020177714.

## Introduction

The world is currently experiencing a pandemic of coronavirus disease (COVID-19) caused by the Severe Acute Respiratory Syndrome coronavirus-2 (SARS-CoV-2) [1]. The risk of morbidity and mortality from the virus is strongly stratified, with poor clinical outcomes considered more likely in certain vulnerable groups. For example, studies from different countries have established that older age groups are at increased risk of death [2, 3].

The ability to identity the population groups most at risk from the virus has manifold public health purposes. Using such data, stratified vaccination policies for governmental delivery can be designed, similar to those for influenza [4]. It may also be possible to prioritise more active monitoring of groups more at risk of clinical deterioration, and facilitate access to healthcare facilities by early identification of the individuals most likely to progress to severe disease who would thus be in need of intensive care and ventilation. Official advice can be issued to vulnerable groups to let them know that they are more at risk from SARS-CoV-2 virus, to promote behaviour modification [5, 6]. Such population groups can also be the target of more formalised ‘segment and shield’ approaches: having divided the population into groups that present with similar health care concerns and needs (segmenting) it is possible to determine which groups require extra protection by reducing interaction with other groups (shielding), whilst relaxing restrictions for the rest of the population [7]. Potential public health policies along this route have been critiqued, however, on an inclusivity basis, particularly due to the unintended harmful consequences to already marginalised groups [8].

In the UK, vulnerable people were stratified into two tiers early on- 30th March 2020 (Table 1); those at risk of severe illness, who were advised to be particularly stringent with social distancing measures, and those within that group at further risk – described as ‘shielded’ individuals – who were advised to self-isolate and were provided with additional advice [9–12]. The former categorisation was based on the groups targeted for National Health Service programmes on influenza vaccination and the latter on clinical consensus. These strata were deliberately broad, to maximise the number of individuals protected. As the evidence evolves – e.g. regarding whether the development of lesions in the cardiovascular system contributes meaningfully to disease pathogenesis in patients with and without pre-existing cardiovascular conditions [13] – there is the opportunity for the categorisation of risk of COVID-19 and serious outcomes from COVID-19 to become more evidence-based.

**Table 1:** UK risk groupings for COVID-19 disease on 30th March 2020

At risk of severe illness		Shielding
Aged 70 or older (regardless of medical conditions)		
Aged under 70 and^a^		
	Chronic (long-term) mild to moderate respiratory diseases, such as asthma, COPD, emphysema or bronchitis	People with severe chest conditions such as cystic fibrosis or severe asthma (requiring hospital admissions or courses of steroid tablets)
	Chronic heart disease, such as heart failure	
	Chronic kidney disease	People with severe diseases of body systems, such as severe kidney disease (dialysis)
	Chronic liver disease, such as hepatitis	
	Chronic neurological conditions, such as Parkinson’s disease, motor neurone disease, MS, a learning disability or cerebral palsy	
	Diabetes	
	A weakened immune system as the result of conditions such as HIV and AIDS, or medicines such as steroid tablets	People who have received an organ transplant and remain on ongoing immunosuppression medication
		People with cancer who are undergoing active chemotherapy or radiotherapy
		People with cancers of the blood or bone marrow such as leukaemia who are at any stage of treatment
	Being seriously overweight (a BMI of 40 or above)	
Those who are pregnant		

Data taken from sources [9–11]. ^a^These groupings represent individuals advised to get a yearly influenza vaccine as an adult for medical reasons. *BMI* body mass index, *COPD* chronic obstructive pulmonary disease, *MS* multiple sclerosis

During epidemics and pandemics of emerging infectious diseases, it is critical to rapidly and accurately identify the populations most at risk. In the case of COVID-19, we undertook a systematic review and quality assessment of the rapidly-evolving global literature in this area, looking at three key outcomes: COVID-19 disease, disease severity, and mortality from the condition. Any potential risk factors, populations, and study designs were included. Arising from our findings, we highlight key knowledge gaps in the current literature and the need for unified global approaches moving forwards, particularly for the next pandemic.

## Materials and methods

### Literature search

We systematically searched Medline, Embase, and Global Health (all via the Ovid platform), in addition to the Web of Science, for published literature between 1st November 2019 and 26th March 2020; then subsequently updated this search for a later period to 29th April. In order to avoid missing publications on risk factors, only terms specific to the virus and the disease were used, which were combined with ‘or’:

- ‘coronavirus’
- ‘covid-19’
- ‘severe acute respiratory syndrome coronavirus 2’
- ‘2019-nCoV-2’
- ‘SARS-CoV-2’
- ‘acute respiratory syndrome’

No limits or filters were applied to the search. The same search terms were used across all databases.

Reference lists of included papers and review articles were also searched, as was the grey literature of public health reports for the 26 countries with the highest numbers of reported patients with COVID-19 at the end of April 2020, for other countries it was assumed there would be insufficient numbers of cases to yield relevant data.

### Eligibility criteria and study selection

The following inclusion and exclusion criteria were applied to the search results.

Inclusion criteria:

- Studies had to provide comparative data on risk factors of any kind for disease (versus no disease), severe disease (versus milder disease) or mortality (versus survival),
- Studies were eligible if they presented data on patients with polymerase chain reaction (PCR)-confirmed SARS-CoV-2 infections. There was considerable variation in case definitions between studies, but PCR testing was the gold standard test for active disease at the start of the pandemic [14], and other testing methods such as Loop-Mediated Isothermal Amplification or serological tests were not included,
- Any study design,
- Any population group,
- Any language of publication.

Exclusion criteria:

- No comparator group included in the study,
- Publication concerned other viruses and diseases,
- Work conducted in animals or in vitro,
- Study population was less than 20 individuals.

Two reviewers independently screened all titles, abstracts and full texts for both literature searches. Discrepancies were resolved by consensus. In all cases where studies were published in any language other than English, with no translations available, these were screened by at least one additional reviewer, with further quality control by another member of the reviewing team.

### Data extraction

Three reviewers independently double-extracted the studies into a pre-designed spreadsheet that collected:

- First author,
- Paper title,
- Journal,
- Type of study,
- Country,
- Study population,
- Overall number in study,
- Number with PCR confirmed SARS-CoV-2,
- Median age of participants/age range,
- Sex ratio,
- Analytical method used,
- Factors adjusted for during the analysis,
- Whether disease, disease severity, or death (or a combination of these) was the outcome of interest,
- The definition of disease severity used, if applicable,
- The risk factors analysed and the direction of effect.

Results were compared and discrepancies resolved by discussion. Data from studies published in languages other than English, at this stage only the Chinese language, were extracted by two additional reviewers, with further quality control by another member of the reviewing team.

### Quality assessment

Two reviewers independently assessed the quality of included studies. Studies published in languages other than English were quality assessed by two additional reviewers, with further quality control by another member of the reviewing team. Assessments were undertaken from the perspective of the objectives of this review, which were not necessarily identical to the objectives of the underlying studies. The quality of included studies was assessed using a checklist adapted from Downs and Black [15], as per the guidance issued by Deeks et al. [16] When assessing the power of studies, the minimum sample size required to detect a relative increase in risk of 10% from a statistically conservative baseline of 50% among the unexposed was calculated at different powers using the Kelsey method within Epi Info, software made available by the United States Center for Disease Control [17]. This 10% value was based on governmental discussions taking place in the UK at the time the review took place. An alpha of 5% was set as the standard. Pragmatically, we assumed only two strata and a ratio of 1:1 between exposure strata. Different thresholds were used for case-control studies and for cohort or cross-sectional studies. These criteria were scored from 0 (< 70% power) to 5 (> 99% power). We considered results sufficient adjusted for confounding if they adjusted for at least the minimal variable set of age, sex, ethnicity and any measure of comorbidities. For ethnically homogenous populations, the need for adjustment for ethnicity was discounted. If two analyses were presented within a single paper with different quality scores, the most conservative score was retained. Studies were not excluded on the basis of the quality assessment.

### Analysis and synthesis

Studies were grouped on the basis of the outcome examined (disease, disease severity, mortality) and then the risk factors examined. Results were classified on the basis of whether they presented evidence as to the exposure under study being a risk factor, taking into account the number of individuals exposed. Where studies focussed on a single risk factor of interest with adjustment for confounding, we extracted all data on potential risks in order to maximise the value of our dataset (whilst accepting that such mutually adjusted estimates for covariates may remain confounded even if that for the primary exposure does not) [18]. As there was substantial heterogeneity in study design, reporting, and the risk factors examined, we present a detailed descriptive summary and narrative synthesis of our findings, rather than a meta-analysis.

### Registration and reporting

This review was registered on PROSPERO as CRD42020177714 and is reported according to the PRISMA guidelines.

## Results

**Fig. 1:**
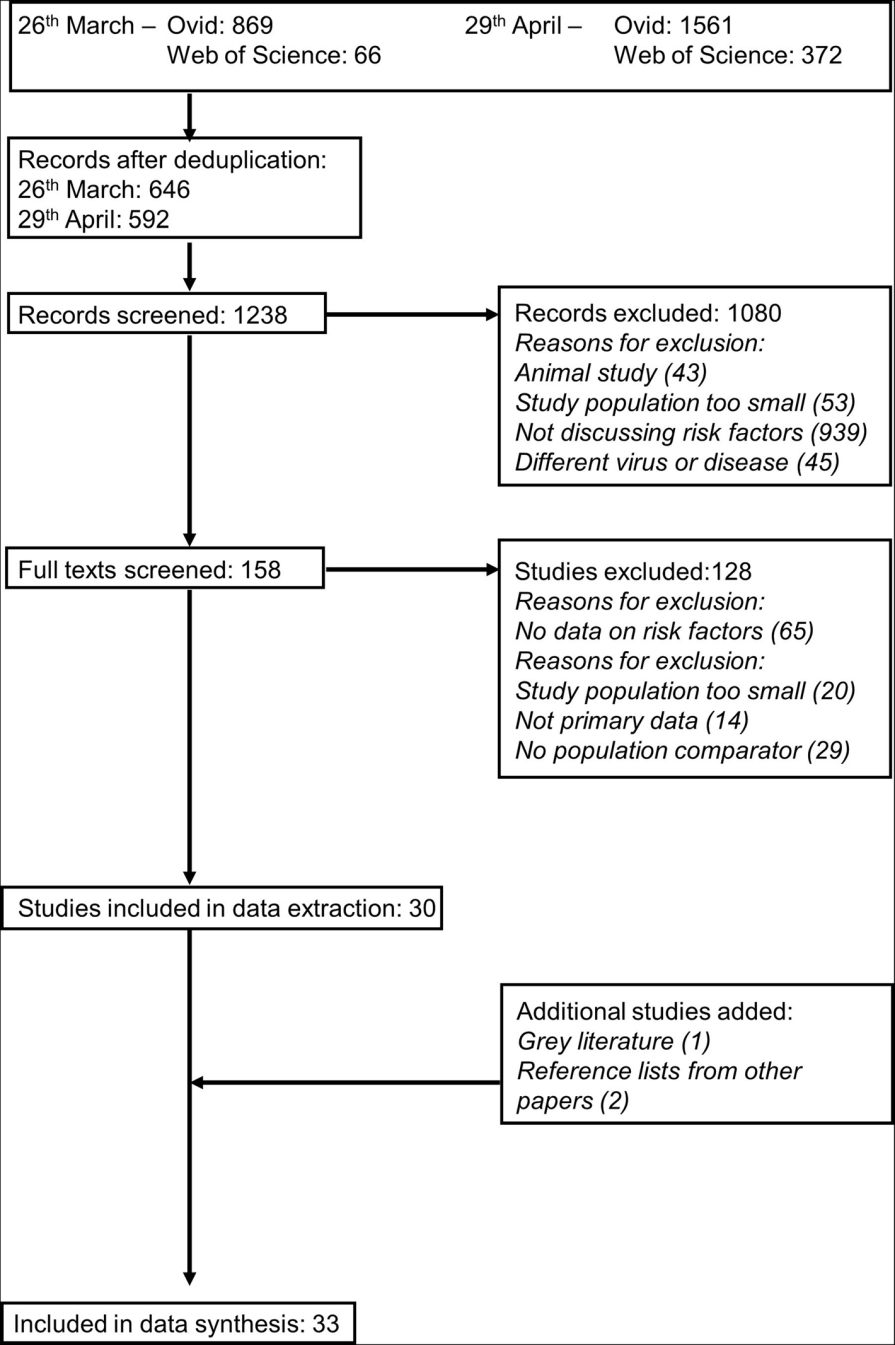
PRISMA flow chart of selection

Two thousand eight hundred and sixty-eight hits were obtained by the searches across the two dates (Fig. 1). After de-duplication across the different databases, this was reduced to 1238. Thirty studies were included at the extraction stage; the main reasons for exclusion were small numbers of participants and studies not having a comparator population. From the grey literature an additional report was included and two studies were identified from reference lists.

Included studies are presented in Table 2. Twenty-nine of the 33 studies were conducted in China, with one each from France, Italy, Singapore and a combined study from England, Wales and Northern Ireland. Six were studies with COVID-19 disease as the outcome, 20 of disease severity and ten of mortality. One additional study looked at a combined outcome of disease severity and mortality.

**Table 2:** Included studies

Author	Type of study	Country	Study population	Overall number of patients	Analytical method used	Factors included in multivariable model	Disease, disease severity, or death	Definition of severity
Cai [19]	Retrospective cohort	China	Hospitalised patients, single hospital	298	Univariable; logistic regression	N/A; age, sex, comorbidities, clinical markers, travel history	Disease severity	As per the international guidelines for community-acquired pneumonia, with a scoring system based on demographics, comorbid illness, physical examination findings, and laboratory and radiographic findings
Chen [20]	Retrospective cohort	China	Hospitalised patients, single hospital	249	Logistic regression	Age, sex, comorbidities and a range of clinical measures	Disease severity	ICU admission
Chen [21]	Retrospective cohort with a nested case-control study	China	Hospitalised patients (moderately, severely or critically ill), single hospital	274	Univariable	N/A	Death	N/A
Chen [22]	Case-control	China	COVID-19 vs. pneumonia negative for SARS-CoV-2	104 (78 COVID-19)	Univariable	N/A	Disease	N/A
Chen [23]	Retrospective cohort	China	Hospitalised patients, single hospital	21	Univariable	N/A	Disease severity	Severe meets any of the following criteria- respiratory distress, RR ≥ 30 breaths/min; SpO_2_ ≤ 93% at rest; and PaO_2_/FIO_2_ ≤ 300. Patients with greater than 50% lesion progression within 24 to 48 h in pulmonary imaging.
Cheng [24]	Case-control	China	Patients presenting with fever diagnosed with pneumonia by specialists with chest CT scans	38 (11 COVID-19)	Univariable	N/A	Disease	N/A
Chinese CDC [25]	Retrospective cohort	China	Nationwide surveillance	72,314	Univariable	N/A	Death	N/A
Fan [26]	Retrospective cohort	Singapore	Hospitalised patients, National Centre for Infectious Diseases	67	Univariable	N/A	Disease severity	ICU admission
Grasselli [27]	Prospective cohort with a nested case-control study	Italy	Hospitalised in ICU across 72 hospitals	1591	Univariable	N/A	Death	N/A
Han [28]	Retrospective cohort	China	Hospitalised patients, single hospital	273	Univariable	N/A	Disease severity	Severe met at least one of the following conditions: a) shortness of breath, RR ≥ 30 times/min, b) oxygen saturation (resting state) ≤93%, or c) PaO_2_/FiO_2_ ≤ 300 mmHg., in addition to positive SARS-CoV-2 RNA nucleic acid test by Reverse transcription polymerase chain reaction, fever, or other respiratory symptoms (the typical CT image abnormities of viral pneumonia were optional). Critical patients also needed to meet at least one of the extra following conditions: a) respiratory failure that needs to receive mechanical ventilation; b) shock; and c) multiple organ failure that need to be transferred to the ICU.
Huang [29]	Retrospective cohort	China	Hospitalised patients, single hospital	41	Univariable	N/A	Disease severity	ICU admission
ICNARC [30]	Case-control (disease) / Prospective cohort (disease severity)	England, Northern Ireland and Wales	Hospitalised patients in ICU across a network of hospitals	12,502 (6720 COVID-19)	Univariable	N/A	Disease; Disease severity	Receiving advanced, as opposed to basic, respiratory support
Liang [31]	Prospective cohort	China	Hospitalised patients across 575 hospitals	1590	Logistic regression; Cox regression	Logistic model adjusted for age, sex, smoking history, comorbidities, cancer	Disease severity, Death	Invasive ventilation, ICU admission, death
Liu [32]	Retrospective cohort	China	Hospitalised patients, single hospital	119	Univariable	N/A	Disease severity	Severe- dyspnoea accompanied by hypoxemia, sometimes acute respiratory distress syndrome, septic shock and multiple organ failure
Liu [33]	Retrospective cohort	China	Hospitalised patients, single hospital	4880	Logistic regression	Age, sex	Disease	N/A
Qiu [34]	Retrospective cohort	China	Hospitalised paediatric patients, three hospitals	36	Univariable	N/A	Disease severity	Moderate disease (mild was baseline)- mild pneumonia; symptoms such as fever, cough, fatigue, headache, and myalgia; no complications and manifestations related to severe conditions
Ruan [35]	Retrospective cohort with a nested case-control study	China	Hospitalised patients across two hospitals with a definitive outcome	150	Univariable	N/A	Death	N/A
Shi [36]	Retrospective cohort	China	Hospitalised patients across a province	487	Univariable; logistic regression	N/A; age, sex, hypertension; full list unknown	Disease severity	Severe pneumonia, characterised by fever, cough, dyspnoea, bilateral pulmonary infiltrates, and acute respiratory injury
Simmonet [37]	Retrospective cohort	France	Hospitalised patients in ICU, single hospital	124	Logistic regression	Age, sex, diabetes, hypertension, BMI	Disease severity	Receiving invasive mechanical ventilation, determined when oxygen therapy (≥ 10 L/min) with target SpO_2_ (90–94%) was ineffective, and when RR was above 25/min, with signs of acute respiratory failure, despite maximal oxygen therapy
Tian [38]	Retrospective cohort	China	Hospitalised individuals transferred from the hospitals of Beijing to the designated hospitals for specialist treatment of infectious diseases by Beijing Emergency Medical Service	262	Univariable	N/A	Disease severity	Severe- dyspnoea or respiratory failure in addition to fever, respiratory symptoms and radiographic evidence of pneumonia
Wan [39]	Prospective cohort	China	Hospitalised patients, single hospital	135	Univariable	N/A	Disease severity	Severe group- respiratory distress, RR ≥ 30 breaths/minute in a resting state, a mean oxygen saturation of ≤93%, and an PaO_2_/FiO_2_ ≤ 300 mmHg
Wang [40]	Case-control	China	Country-wide data compared to historic SARS/MERS	11,425 (835 COVID-19)	Univariable	N/A	Disease	N/A
Wang [41]	Retrospective cohort	China	Hospitalised patients, single hospital	138	Univariable	N/A	Disease severity	ICU admission
Wang [42]	Prospective cohort	China	Hospitalised patients, single hospital	116	Univariable	N/A	Disease severity	Severe- fever or suspected respiratory infection, plus one of the following: RR > 30 breaths/min; severe respiratory distress; or SpO_2_ ≤ 93% on room air
Wang [43]	Cross-sectional	China	Hospitalised patients, single hospital	69	Univariable	N/A	Disease severity	SpO_2_ < 90%
Wu [44]	Retrospective cohort	China	Hospitalised patients, single hospital	201	Univariable; Cox regression	N/A	Disease severity, Death	ARDS, mechanical ventilation
Wu [45]	Prospective cohort	China	Country-wide data	44,672	Univariable	N/A	Death	N/A
Yang [46]	Retrospective cohort	China	Hospitalised patients, single hospital, admitted to ICU	52	Univariable; Cox regression	N/A	Death	N/A
Yuan [47]	Retrospective cohort	China	Hospitalised patients, single hospital	27	Univariable	N/A	Death	N/A
Zhang [48]	Retrospective cohort	China	Hospitalised patients, single hospital	140	Univariable	N/A	Disease severity	Severe COVID-19 was designated when the patients had one of the following criteria: (a) respiratory distress with respiratory frequency ≥ 30/min; (b) pulse oximeter oxygen saturation ≤ 93% at rest; and (c) oxygenation index (PaO_2_/FiO_2_) ≤ 300 mmHg.
Zhang [49]	Retrospective cohort	China	Hospitalised patients (patients with no severe underlying diseases), single hospital	95	Univariable	N/A	Disease severity	Severe- RR ≥ 30 times / min; at rest, oxygen saturation ≤ 93%; PaO_2_/FiO_2_ ≤ 300 mmHg
Zhang [50]	Retrospective cohort	China	Hospitalised patients, single hospital	120	Univariable	N/A	Disease severity	Severe- when patients met one of the following criteria: (1) respiratory distress with a breathing rate ≥ 30/min; (2) pulse oximeter oxygen saturation ≤ 93% at rest; (3) oxygenation index (PaO_2_/FiO_2_) ≤300 mmHg; (4) respiratory failure requiring mechanical ventilation; (5) shock; and (6) combined with other organ failure requiring ICU monitoring and treatment
Zhou [51]	Retrospective cohort with a nested case-control study	China	Hospitalised patients across two hospitals with a definitive outcome	191	Univariable; logistic regression	N/A; age, coronary heart disease, Sequential Organ Failure Assessment score, lymphocyte count, D-dimer	Death	N/A

*ARDS* acute respiratory distress syndrome, *BMI* body mass index, *CDC* Center for Disease Control and Prevention, *CT* computed tomography, *FiO*_*2*_ inspired oxygen fraction, *ICNARC* intensive care national audit and research centre, *ICU* intensive care unit, *MERS* Middle Eastern Respiratory Syndrome, *N/A* not applicable, *PaO*_*2*_ arterial partial pressure of oxygen, *RR* respiratory rate, *SARS* severe acute respiratory syndrome, *SpO*_*2*_ oxygen saturation

### Quality assessment

Included studies were generally too small to detect a 10% increase in risk of disease, disease severity, or mortality (Table 3). One study among the 33 was assessed to have 95% power and two others 99%; all were large, national, investigations. As 26 studies were purely descriptive or presented univariable analysis only, there was no adjustment for confounding. Remaining studies with a regression component did not adjust for our minimal confounder set. Only nine studies provided estimates of the random variability of effect estimates. The majority of studies ascertained exposure information from clinical records, which would have collected data prospectively and thus with limited recall bias. Blinding of outcome and exposure recording by investigators was not documented. In the case of certain disease severity outcomes, such as admittance to intensive care units (ICU), variability in thresholds for reaching these outcomes is likely to exist between settings and clinicians.

**Table 3:** Quality assessment

Author	Is the study design clearly reported?	Is the hypothesis/aim/objective of the study clearly described?	Are the main outcomes to be measured clearly described in the Introduction or Methods section?	Are the characteristics of the patients included in the study clearly described?	Are the distributions of principal confounders in each group of subjects to be compared clearly described?	Are the main findings of the study clearly described?	Does the study provide estimates of the random variability in the data for the main outcomes?	Have actual probability values been reported for the main outcomes except where the probability value is < 0.001?	Was there potential for recall bias in the ascertainment of the exposure?	Was there potential for differential or non-differential misclassification of the exposure?	Was there potential for observer bias in ascertainment of the outcome?	Was there potential for differential or non-differential misclassification of the outcome?	If any of the results of the study were based on ‘data dredging’ was this made clear?	Do the analyses adjust for different lengths of follow-up of patients, or in case-control studies, is the time period between the intervention and outcome the same for cases and controls?	Were the statistical tests used to assess the main outcomes appropriate?	Were the main outcome measures used accurate (valid and reliable)?	Were the patients in different intervention groups (trials and cohort studies) or were the cases and controls (case-control studies) recruited from the same population?	Were study subjects in different intervention groups (trials and cohort studies) or were the cases and controls (case-control studies) recruited over the same period of time?	Was there adequate adjustment for confounding in the analyses of interest?	Were losses of patients to follow-up taken into account?	Are the study results appropriately interpreted e.g. in terms of the strength of the evidence, its application/implications, causality?	Did the study have sufficient power to detect a clinically important effect where the probability value for a difference being due to chance is less than 5%?^a^
Cai [19]	Y	Y	Y	Y	Y	Y	Y	Y	N	N	Y	Y	N/A	N	Y	Y	Y	Y	Y	U	Y	0
Chen [20]	Y	Y	Y	Y	N	Y	Y	Y	N	U	N	Y	N/A	N	Y	Y	Y	Y	N	U	Y	0
Chen [21]	Y	Y	Y	Y	N	Y	N	N	Y	Y	N	N	N/A	N	N/A	Y	Y	Y	N	U	N	0
Chen [22]	Y	Y	Y	Y	N	Y	N	Y	N	N	N	N	N/A	N	Y	Y	Y	Y	N	U	Y	0
Chen [23]	Y	Y	Y	Y	N	Y	N	Y	N	U	Y	Y	N/A	N	Y	U	Y	Y	N	U	Y	0
Cheng [24]	Y	Y	Y	Y	N	Y	N	Y	N	N	Y	Y	N/A	N	Y	Y	Y	Y	N	U	N	0
Chinese CDC [25]	Y	Y	Y	Y	Y	Y	N	Y	Y	Y	Y	N	N/A	Y	Y	Y	Y	Y	N	U	Y	5
Fan [26]	Y	Y	Y	Y	N	Y	N	Y	N	N	Y	Y	N/A	N	Y	Y	Y	Y	N	U	Y	0
Grasselli [27]	Y	Y	Y	Y	N	Y	Y	Y	N	N	Y	Y	N/A	N	Y	Y	Y	Y	N	U	Y	0
Han [28]	Y	Y	Y	Y	N	Y	N	N	N	N	N	N	N/A	N	N/A	U	Y	Y	N	U	Y	0
Huang [29]	Y	Y	Y	Y	N	Y	N	Y	Y	Y	Y	Y	N/A	N	Y	Y	Y	Y	N	U	Y	0
ICNARC [30]	Y	Y	N	Y	Y	Y	N	N	U	U	U	U	N/A	N	N/A	U	N	N	N	U	Y	4
Liang [31]	Y	Y	Y	Y	N	Y	Y	Y	N	Y	Y	Y	N/A	Y	Y	Y	Y	Y	Y	Y	Y	0
Liu [32]	Y	Y	Y	Y	N	Y	N	N	N	N	Y	Y	N/A	N	Y	U	Y	Y	N	U	Y	0
Liu [33]	Y	Y	Y	Y	N	Y	Y	Y	U	U	N	N	N/A	N	Y	Y	Y	Y	N	U	Y	3
Qiu [34]	Y	Y	Y	Y	N	Y	N	Y	N	N	Y	Y	N/A	N	Y	U	Y	Y	N	U	Y	0
Ruan [35]	Y	Y	Y	Y	N	Y	N	Y	N	N	Y	Y	N/A	N	Y	Y	Y	Y	N	U	Y	0
Shi [36]	Y	Y	Y	Y	N	N	Y	Y	N	U	U	U	N/A	N	Y	U	Y	Y	N	U	Y	0
Simmonet [37]	Y	Y	Y	Y	N	Y	Y	Y	N	N	Y	Y	N/A	N	Y	Y	Y	Y	N	U	Y	0
Tian [38]	Y	Y	Y	Y	N	Y	N	Y	N	N	Y	Y	N/A	N	Y	U	Y	Y	N	U	Y	0
Wan [39]	Y	Y	Y	Y	N	Y	N	Y	N	N	Y	Y	N/A	N	Y	Y	Y	Y	N	U	Y	0
Wang [40]	Y	Y	Y	Y	N	Y	N	N	N	N	N	N	N/A	N	N/A	Y	N	N	N	U	Y	0
Wang [41]	Y	Y	Y	Y	N	Y	N	Y	N	U	Y	Y	N/A	N	Y	Y	Y	Y	N	U	Y	0
Wang [42]	Y	Y	Y	Y	N	Y	N	Y	N	U	Y	Y	N/A	N	Y	U	Y	Y	N	U	Y	0
Wang [43]	Y	Y	Y	Y	N	Y	N	Y	N	U	N	Y	N/A	N	Y	Y	Y	Y	N	U	Y	0
Wu [44]	Y	Y	Y	Y	N	Y	Y	Y	N	U	N	Y	N/A	Y	Y	U	Y	Y	N	Y	Y	0
Wu [45]	Y	Y	Y	Y	N	Y	N	N	U	U	Y	Y	N/A	N	N/A	Y	Y	Y	N	U	Y	5
Yang [46]	Y	Y	Y	Y	N	Y	N	N	N	U	N	Y	N/A	N	N/A	U	Y	Y	N	U	Y	0
Yuan [47]	Y	Y	Y	Y	N	Y	N	Y	N	U	N	N	N/A	N	Y	Y	Y	Y	N	U	Y	0
Zhang [48]	Y	Y	Y	Y	N	Y	N	Y	N	U	N	N	N/A	N	Y	Y	Y	Y	N	U	Y	0
Zhang [49]	Y	Y	Y	Y	N	Y	N	Y	N	N	N	N	N/A	N	Y	Y	Y	Y	N	U	N	0
Zhang [50]	Y	Y	Y	Y	N	Y	N	Y	U	U	N	Y	N/A	N	Y	U	Y	Y	N	U	Y	0
Zhou [51]	Y	Y	Y	Y	N	Y	Y	Y	N	U	N	N	N/A	N	Y	Y	Y	Y	N	U	Y	0

^a^0 = < 70% power, 1 = 70%, 2 = 80%, 3 = 90%, 4 = 95%, 5 = 99%. *CDC* Center for Disease Control Prevention, *ICNARC* intensive care national audit and research centre, *N/A* not applicable

### Risk factors for disease

Six studies compared the likelihood of having COVID-19 to other infectious conditions (Table 4). Of note, as testing strategies were largely focussed on hospitalised individuals i.e. those displaying noticeable symptoms, studies were of the likelihood of COVID-19 disease, rather than more broadly of SARS-CoV-2 infection (and particularly of severe disease, although patients with mild and symptomatic infection were also reported to be hospitalised in some studies for the purposes of isolation or observation). Age and sex were key foci as potential risk factors, comparing patients with COVID-19 to either: a) SARS-CoV or Middle Eastern Respiratory Syndrome (MERS), or b) other forms of pneumonia. Generally, sex ratios were skewed such that men were over-represented among those with disease. In England, Northern Ireland, and Wales, Asian and Black individuals were found to be at increased risk of COVID-19 in descriptive analyses, with 15.4 and 10.7% of patients falling into these groupings, respectively, versus 5.8 and 2.8% of individuals with other viral pneumonia [30]. Higher body mass index (BMI) was also suggested to be a risk factor with two descriptive analyses, for example in the Intensive Care National Audit and Research Centre (ICNARC) report 31.2% of COVID-19 patients had a BMI of 30- < 40, versus 23.5% of people with other viral pneumonia [30, 37]. Given the large, national, scope of the ICNARC dataset, results from it are particularly likely to be reliable.

**Table 4:** Potential risk factors for disease

Potential risk factor	Study supports risk	Study does not support risk or is neutral
Age	Patients older than those with COVID-19 and younger than those with MERS (descriptive) [40] Younger ages (median 45 years) in patients with COVID-19 than other pneumonia (median 61) (statistical test) [22] Increasing risk of positivity for SARS-CoV-2 among COVID-19 suspects (on the basis of symptoms/contact tracing) with age (odds ratio 1.02) but unclear categorisation of age (multivariable regression) [33]	Median age 60 years in those with COVID-19 and 61 in those with other viral pneumonia (descriptive) [30] Mean age 50 years in COVID-19 patients vs. 44 in individuals with other pneumonia (statistical test) [24]
Sex	Sex ratio skewed towards men for COVID-19, akin to MERS but not SARS (descriptive) [40] Sex ratio skewed towards men for COVID-19 versus other viral pneumonia (descriptive) [30] Greater proportion male in COVID-19 versus other pneumonia, although small sample size and thus low statistical certainty (statistical test) [24] Increasing risk of positivity for SARS-CoV-2 among COVID-19 suspects (on the basis of symptoms/contact tracing) among males versus female (odds ratio 1.16) (logistic regression) [33]	Sex distribution similar amongst patients with COVID-19 and other pneumonia (statistical test) [22]
Ethnicity	Higher percentage of Black and Asian individuals amongst COVID-19 patients than patients with other viral pneumonias (descriptive) [30]	
Index of multiple deprivation		Distribution of deprivation similar across COVID-19 and other viral pneumonia (descriptive) [30]
Body mass index	Greater proportion of COVID-19 patients had higher body mass index than individuals with other pneumonia (descriptive) [37] Greater proportion of COVID-19 patients had higher body mass index than individuals with other viral pneumonia (descriptive) [30]	
Pregnancy		Percentage of women who were pregnant similar across COVID-19 and other viral pneumonia (descriptive) [30]

*MERS* middle eastern respiratory syndrome, *SARS* severe acute respiratory syndrome

### Risk factors for severe disease

Among the 20 studies of risk factors for severe versus milder disease and one of a mixed outcome (severe disease and death), a wide array of definitions of severity were used, such as ICU admission, the need for mechanical ventilation, and various measures of respiration and oxygenation (Table 2). Many risk factors were examined (Table 5). As well as potential demographic risks (age, sex, ethnicity), behavioural traits (smoking) and broad clinical factors (BMI, infectious diseases) were analysed. Large numbers of papers sought to explore the implications of different comorbidities on the risk of severe COVID-19, particularly respiratory and cardiovascular conditions.

**Table 5:** Potential risk factors for disease severity

Potential risk factor	Study supports risk	Study does not support risk or is neutral
Sex	Odds ratio for severe disease 3.68 for men compared to women (multivariable regression) [36] Odds ratio for invasive mechanical ventilation 2.83 for men compared to women (multivariable regression) [37] Females less likely to be admitted to ICU, require mechanical ventilation, or die; odds ratio 0.61 (multivariable logistic regression) [31]	Sex distribution similar in severe and non-severe disease (descriptive) [30, 49] Sex distribution similar in severe and non-severe disease (statistical test) [23, 26, 28, 29, 32, 34, 38, 39, 41–44, 48–50] Sex distribution similar in severe and non-severe disease (multivariable regression) [19, 20]
Age	Average^a^ 61 years severe disease, 45 otherwise (statistical test) [38] Average^a^ 61 years severe disease, 52 years moderate disease (statistical test) [23] Average^a^ 56 years severe disease, 44 years mild disease (statistical test) [39] Median 67 years acute respiratory distress syndrome, 52 severe, 45 mild (statistical test) [42] Median 64 years severe patients, 52 years otherwise (statistical test) [48] Mean 61 years severe, otherwise 40 (statistical test) [50] Median 71 years SpO_2_ < 90%, 37 years SpO_2_ ≥ 90% (statistical test) [43] Median 66 years patients in ICU, 51 otherwise (statistical test) [41] Median 54 years patients in ICU, 41 otherwise (statistical test) [26] 65 years and over 3.26 times the hazard rate of ARDS than those under 65 (univariable regression) [44] Age associated with ICU admission, odds ratio 1.06 but unclear for what categorisation of age (multivariable regression) [20]	Distribution of age did not differ by disease severity (descriptive) [30, 34, 49] Distribution of age did not differ by disease severity (statistical test) [28, 29, 32] Confidence interval for effect of age (categorisation unclear) crosses the null (multivariable regression) [37]
	Mean 56 years severe disease, 45 years mild disease; odds ratio 1.06 but unclear categorisation of age (multivariable regression) [36] Mean 63 years severe disease, 41 years mild disease; odds ratio 1.08 but unclear categorisation of age (multivariable regression) [19] Older individuals more likely to be admitted to ICU, require mechanical ventilation, or die; odds ratio 1.05, categories of age unclear (multivariable logistic regression) [31]	
Ethnicity	76.3% of individuals receiving basic respiratory support were White versus 65.6% receiving advanced respiratory support; Asian and Black ethnicities appear most at risk of severe disease (England, Northern Ireland and Wales; descriptive) [30]	Distribution of disease severity similar across ethnic groups (Chinese, Malay, Indian, other – with small numbers in groups other than Chinese; study in Singapore; descriptive) [26]
Deprivation		Distribution across deprivation categories similar (descriptive) [30]
Pregnancy		Distribution in pregnant and non-pregnant individuals similar across disease severity (descriptive) [30]
Smoking	100% of current smokers had severe disease, but only six individuals smoked [50]	Distribution in current and non-current smokers similar across disease severity (descriptive), only three individuals smoked [39] Distribution in current and non-current smokers similar across disease severity (statistical test); small numbers who smoked [29] Distribution in historical/current and non-smokers similar across disease severity (statistical test) [36, 48]
Body mass index	≥35 kg/m^2^ risk factor versus < 25 kg/m^2^ for invasive mechanical ventilation; odds ratio 7.36. Results for other strata cross the null (multivariable regression) [37] Increasing body mass index increased risk; odds ratio 1.17 (categorisation unclear) [19]	Distribution of disease severity similar across body mass index categories (descriptive) [30]
Any/other comorbidity	Presence of comorbidity more common among those with severe disease (statistical test) [39, 41, 48, 50]	Distribution with and without condition similar across disease severity (statistical test) [23, 29, 36] Distribution with and without comorbidities not otherwise considered in the study similar across disease severity (statistical test) [50] Distribution with and without condition similar across disease severity (multivariable regression) [20]
Cardiovascular disease/chronic heart disease/coronary heart disease	Presence of comorbidity more common among those with severe disease (descriptive) [39] Presence of comorbidity more common among those with severe disease (statistical test) [19, 36, 41, 43, 50]	Distribution with and without condition similar across disease severity (descriptive) [30, 44] Distribution with and without condition similar across disease severity (statistical test) [29, 48]
Hypertension	Presence of comorbidity more common among those with severe disease (statistical test) [41, 43, 50] Hazard ratio of ARDS 1.82 in those with the condition versus those without (univariable regression) [44] Odds ratio of severe disease 2.71 in those with the condition versus those without (multivariable regression) [36] Odds ratio of being admitted to ICU, require mechanical ventilation, or die 1.89 in those with the condition versus those without (multivariable regression) [31]	Distribution with and without condition similar across disease severity (descriptive) [39] Distribution with and without condition similar across disease severity (statistical test); one study with small numbers with the condition [23, 29, 42, 48] Confidence interval in presence and absence of condition crosses the null (multivariable regression) [19] Confidence interval in presence and absence of condition crosses the null (multivariable regression, result borderline) [37]
Diabetes	Presence of comorbidity more common among those with severe disease (descriptive) [39] Presence of comorbidity more common among those with severe disease (statistical test) [19, 36, 41, 43, 50] Hazard ratio of ARDS 2.34 in those with the condition versus those without (univariable regression) [44] Odds ratio of being admitted to ICU, require mechanical ventilation, or die 2.21 in those with the condition versus those without (multivariable regression) [31]	Distribution with and without condition similar across disease severity (statistical test); small numbers with condition [23] Distribution with and without condition similar across disease severity (statistical test) [29, 48] Distribution with and without condition similar across disease severity (statistical test, borderline result) [42] Confidence interval in presence and absence of condition crosses the null (multivariable regression) [37]
Respiratory/pulmonary disease		Distribution with and without condition similar across disease severity (descriptive) [30, 39]
Asthma		Distribution with and without condition similar across disease severity (statistical test); small numbers with condition [43]
Chronic obstructive pulmonary disease (COPD)	Presence of comorbidity more common among those with severe disease (descriptive); small numbers with condition [39] Presence of comorbidity more common among those with severe disease (statistical test); both studies have small numbers with the condition [41, 50] Odds ratio of being admitted to ICU, require mechanical ventilation, or die 3.40 in those with the condition versus those without (multivariable regression) [31]	Distribution with and without condition similar across disease severity (statistical test); small numbers with condition [29, 43, 48]
Pulmonary tuberculosis		Distribution with and without condition similar across disease severity (statistical test); small numbers with condition [48]
Malignancy	Presence of comorbidity more common among those with severe disease (statistical test); small numbers with condition [39] Presence of comorbidity more common among those with severe disease (statistical test) [36, 42, 50] Presence of comorbidity more common among those with severe disease (multivariable analysis) [31]	Distribution with and without condition similar across disease severity (descriptive) [30] Distribution with and without condition similar across disease severity (statistical test) [41] Distribution with and without condition similar across disease severity (statistical test); small numbers with condition [19, 29, 43]
Cerebrovascular disease	Presence of comorbidity more common among those with severe disease (statistical test) [41]	
Arrhythmia		Distribution with and without condition similar across disease severity (statistical test); small numbers with the condition [48]
Cerebral infarction		Distribution with and without condition similar across disease severity (statistical test) [42]
Stroke		Distribution with and without condition similar across disease severity (statistical test); small numbers with condition [48]
Aorta sclerosis		Distribution with and without condition similar across disease severity (statistical test); small numbers with condition [48]
Chronic kidney disease/renal issues	Presence of comorbidity more common among those with severe disease (statistical test) [42]	Distribution with and without condition similar across disease severity (descriptive) [30] Distribution with and without condition similar across disease severity (statistical test); small numbers with the condition [41]
Chronic renal disease/insufficiency		Distribution with and without condition similar across disease severity (statistical test); one study has small numbers of patients with the condition [36, 48]
Chronic liver disease		Distribution with and without condition similar across disease severity (descriptive), sometimes small numbers with condition [19, 30, 39] Distribution with and without condition similar across disease severity (statistical test) [36, 41] Distribution with and without condition similar across disease severity (statistical test); small numbers with condition [29, 50]
Fatty liver and abnormal liver function		Distribution with and without condition similar across disease severity (statistical test) [48]
Hyperlipidaemia		Distribution with and without condition similar across disease severity (statistical test) [48]
Dyslipidemia		Confidence interval in presence and absence of condition crosses the null (multivariable regression) [37]
Chronic gastritis/gastric ulcer		Distribution with and without condition similar across disease severity (statistical test) [48]
Cholelithiasis		Distribution with and without condition similar across disease severity (statistical test) [48]
Urolithiasis		Distribution with and without condition similar across disease severity (statistical test); small numbers with condition [48]
Thyroid diseases		Distribution with and without condition similar across disease severity (statistical test); small numbers with the condition [48]
Electrolyte imbalance	Presence of comorbidity more common among those with severe disease (statistical test); small numbers with condition [48]	
Agglomerative disease		Distribution with and without condition similar across disease severity (descriptive); small numbers with the condition [39]
Immunocompromised		Distribution with and without condition similar across disease severity (descriptive) [30]
Chronic hepatitis		Distribution with and without condition similar across disease severity (statistical test); small numbers with condition [43]
HIV		Distribution with and without condition similar across disease severity (statistical test); small numbers with condition [41]
Living without assistance		Distribution with and without condition similar across disease severity (descriptive) [30]

One study included death in a combined measure of disease severity [31]. ^a^Unclear as to whether mean, median or mode. *ARDS* acute respiratory distress syndrome, *ICU* intensive care unit, *SpO*_*2*_ oxygen saturation

The least equivocal evidence was presented for age as a risk factor, including four studies where it was an independent risk in a multivariable regression model [19, 20, 31, 36]. The clearest analysis to present age data (i.e. which used different comparison groups) was a univariable regression model where individuals 65 years and over had 3.26 times the hazard rate of ARDS than those under 65 [44]. Eight studies suggested that diabetes could be a risk factor [19, 31, 36, 39, 41, 43, 44, 50], six hypertension [31, 36, 41, 43, 44, 50], and four the presence of unspecified comorbidities) [39, 41, 48, 50], but the balance of evidence for these co-morbidities being risk factors was generally inconclusive. Many other factors were examined by one study, often with small numbers of individuals with the condition. None of the included studies for disease severity were assessed to have been powered to detect a 10% increase in effect size.

### Risk factors for mortality

Ten studies examined risk factors for mortality, often by nesting case-control studies within prospective or retrospective cohorts (Table 6). Among these studies, many included statistical testing, but none presented an adjusted regression model for the risk factors considered.

**Table 6:** Potential risk factors for mortality

Potential risk factor	Study supports risk	Study does not support risk or is neutral
Sex	Men more at risk (descriptive) [21]	Sex distribution similar amongst patients who died and survived (descriptive) [25, 35, 46, 47] Confidence interval for males versus females crosses the null (univariable regression) [44]
Age	Over 60 years particularly at risk (descriptive) [21] 8% case fatality ratio in 70–79 year olds and 14.8% in those over 80. Overall figure 2.3% (descriptive) [45] Median age in those who died 52 years, 65 years among survivors (descriptive) [46] Over 50 years of age particularly at risk- 1.3% died 50–59 years, 3.6% 60–69 years, 8.0% 70–79 years, 14.8% 80 years plus; less than 1% all other age groups (descriptive) [25] Risk begins to increase at approximately 50 years (statistical test, but graphical presentation) [35] Median age in those who died 68 years, among those who survived 55 (statistical test) [47] Over 61 years, increasing per 10 year age group (statistical test) [27] 65 years and older 6.17 the hazard rate of those under 65 (univariable regression) [44]	
Smoking		Proportion of smokers similar among those who died versus those who did not (descriptive); one study had small numbers of smokers [21, 46] Distribution of current smokers similar among survivors and non-survivors (univariable regression analysis, not included in multivariable model) [51]
Pregnancy		Proportion of women who were pregnant similar amongst patients who died versus survived (descriptive) [21]
Any comorbidity	Presence of any comorbidity more common among those dying (descriptive) [21, 35, 46, 47, 51]	
Hypertension	Presence of condition more common among those dying (descriptive) [3, 21, 25, 27] Presence of condition more common among those dying (statistical test) [47, 51]	Confidence interval for individuals with and without the condition crosses the null (univariable regression) [44]
Cardiovascular disease/chronic heart disease	Presence of condition more common among those dying (descriptive) [21, 25, 35, 45] Presence of condition more common among those dying (statistical test) [47, 51]	Distribution dying in presence and absence of comorbidity similar (descriptive), sometimes small numbers with the condition [44, 46]
Diabetes	Presence of condition more common among those dying (descriptive) [21, 25, 45, 46] Presence of condition more common among those dying (statistical test) [47, 51]	Confidence interval for individuals with and without the condition crosses the null (univariable regression) [44]
Chronic respiratory/lung disease (chronic obstructive lung disease)	Presence of condition more common among those dying (descriptive) [21, 45] Presence of condition more common among those dying (statistical test) [51]	Distribution dying in presence and absence of comorbidity similar (descriptive) [46]
Respiratory infectious disease	Presence of condition more common among those dying (descriptive) [25]	
Malignancy	Presence of condition more common among those dying (descriptive) [3, 25]	Distribution dying in presence and absence of comorbidity similar (descriptive), sometimes small numbers with the condition [46, 47, 51]
Cerebral infarction/ cerebrovascular disease	Presence of condition more common among those dying (descriptive) [46]	Distribution dying in presence and absence of comorbidity similar (statistical test); small numbers with the condition [47]
Chronic gastritis		Distribution dying in presence and absence of comorbidity similar (statistical test); small numbers with the condition [47]
Chronic kidney disease	Presence of condition more common among those dying (statistical test); small numbers with the condition [51]	
Dementia		Distribution dying in presence and absence of comorbidity similar (descriptive); small numbers with the condition [46]
Malnutrition		Distribution dying in presence and absence of comorbidity similar (descriptive); small numbers with the condition [46]
Hepatitis B virus infection		Distribution dying in presence and absence of comorbidity similar (descriptive) [21]

Eight studies examined age and all provided evidence for it being a risk factor for mortality [21, 25, 27, 35, 44–47], although none adjusted for other factors, such as comorbidities. Age groups from 50 upwards were considered particularly at risk. In the single regression analysis, the hazard rate for death in those 65 years or over was estimated to be six times that of individuals under 65 [44]. The evidence was similarly consistent for general comorbidities (albeit all the studies were descriptive); among individuals who died, comorbidities were 1.5 to 2.8 times more common than among those who survived [21, 35, 46, 47, 51]. Specific comorbidities were discussed in several studies, generally under overarching classifications such as ‘cardiovascular disease’ or ‘diabetes’, with more specific definitions not provided. Evidence was more equivocal, but still in favour, of hypertension [3, 21, 25, 27, 47, 51], cardiovascular disease [21, 25, 35, 45, 47, 51], diabetes [21, 25, 45–47, 51], and chronic respiratory/lung diseases being risk factors (references presented for studies in support only) [21, 45, 51]. Of these studies, data from two well-powered, national-level studies from China supported cardiovascular disease and diabetes as risk factors for mortality from COVID-19 [25, 45].

## Discussion

In this systematic review of risk factors for COVID-19 disease, disease severity and mortality, we document 33 comparative studies examining sociodemographic, behavioural and clinical exposures. Age and sex were very commonly examined; a wide array of comorbidities have also been considered.

Within the synthesised evidence, risk factors for mortality were the clearest, plausibly partly because this outcome is easy to define. Increasing age (different studies presented different thresholds, but being over 50 years of age was common) was an uncontested risk factor. Five studies also presented evidence for the presence of any comorbidities being a risk factor [21, 35, 46, 47, 51], with none demonstrating evidence against. Given the increasing prevalence of comorbidities with age, the lack of adjustment for confounding in these studies likely over-emphasises the effect size of each risk factor. We note that work subsequent to our literature search documents an independent effect of age on COVID-19 mortality from overall comorbidities, as measured by the Charlson Comorbidity Index Score, but not vice-versa [52]. Another study published outside of the time range of our search found both age and an array of comorbidities, each analysed separately (chronic cardiac disease, chronic pulmonary disease, chronic kidney disease, chronic neurological disease, dementia, malignancy, moderate/severe liver disease; and obesity), to be independent risk factors (as well as sex) [53].

Risk factors for severe disease were more complex to synthesise, likely due to the mixed array of outcome measures that can also be prone to observer bias. The impact of age was very commonly assessed, generally showing evidence in favour of this being a risk factor (with a similar age spectrum to the mortality data). Ethnicity was studied in two publications internationally [26, 30], with mixed results. We note that such findings are likely to be highly context-specific, given that ethnicity acts as a proxy for a series of sociodemographic factors that are highly relevant to the spread of an infectious condition (as well as, perhaps, some biological traits).

Studies of risk factors for COVID-19 disease have been complicated by testing strategies globally, which have largely been concentrated on severe disease. As our knowledge of the full symptom spectrum of the disease moves forward, it will be possible to have a broader case definition that does not solely focus on viral testing, and thus the ability for more generalised complementary studies. Additionally, serological surveys assessing the history of infection with SARS-CoV-2 in different population groups will allow the identification of risk factors for infection, whether symptomatic or not. Both ethnicity (Black and Asian individuals at higher risk; from a single study in England, Northern Ireland and Wales) [30] and higher BMI were found to be associated with disease severity within the included literature [30, 37], again from descriptive studies only. While these studies were not eligible for our review, we note a series of reports from non-comparative studies documenting the potential influence of ethnicity on the likelihood of getting COVID-19 e.g. the work of Price-Haywood from the US [52]. Male sex was reasonably consistently shown to be a risk factor for presence of COVID-19 but not with severity of disease or mortality [24, 30, 40]. As with ethnicity, socioeconomic and behavioural factors make this association likely to vary between settings.

In considering the role of comorbidities in COVID-19, it is important to consider the underlying pathology of the virus. Respiratory coronaviruses associated with the common cold in immunocompetent people generally affect only cells in the upper respiratory tract (URT), whereas the previously discovered highly pathogenic coronaviruses SARS-CoV and MERS-CoV affect cells in the URT and lower respiratory tract (LRT). SARS-CoV-2 has been shown to do the same [54], and one of the host cell receptors it targets is Angiotensin-Converting Enzyme 2 (ACE2), with a second major receptor being Transmembrane Serine Protease 2 (TMPRSS2) [55]. SARS-CoV-2 can infect all the major cell types in the respiratory tract – type I and type II pneumocytes, alveolar macrophages and endothelial cells [56, 57]. This infection leads to cell death, with significant leaking of fluid into the alveolar spaces (pulmonary oedema), which compromises gas exchange [58], eventually leading to ARDS. The inflammatory response adds aggregation of repair proteins such as fibrin, which can lead to creation of hyaline membranes which further reduces the surface available for gas exchange [58]. Subsequently, inflammatory cells are activated, recruited by release or exposure of cytokines such as the interleukins (IL) 1β and 6, monocyte chemoattractant protein-1 [56], and proteins of the extracellular matrix, as well as upregulation of the complement system. Inflammatory cells release cytokines which have systemic effects, eventually leading to disseminated intravascular coagulation (DIC), hypotensive shock and metabolic disturbances if not checked [58].

This pathogenesis therefore offers several points where co-morbidities may exacerbate the process. The target receptor TMPRSS2 is modulated in response to air pollution and in autoimmune conditions such as asthma [55], which may affect the number of receptors available for SARS-CoV-2 to target, and ACE2 is involved in the renin-angiotensin system (RAS) which controls blood pressure. Viral interference causes dysfunction, which leads to a pro-inflammatory state and increased vascular permeability in response to changes in vascular contraction and sodium homeostasis – exacerbating the effect from the physical damage to the affected cells [58]. Conditions causing hypertension – both primary and secondary to renal disease, endocrine dysfunctions such as hypothyroidism, cardiovascular dysfunction such as arteriosclerosis, or neurological dysfunctions such as acute stress – also affect the RAS [58], meaning that these conditions might be expected to exacerbate pathology caused by SARS-CoV-2. Any condition creating a pro-inflammatory state, such as type II diabetes or pre-existing infection, or involving autoimmunity, such as type I diabetes, might also be expected to contribute to increased pathology. There is also the direct effect of cell damage – if the target tissues are already damaged this reduces ‘spare’ capacity and therefore the leeway for adaptation to allow the host to continue to maintain homeostasis whilst still being able to eliminate the pathogen and repair the damage. The need for inflammatory cells to clear the infection is also a potential area of interface with comorbidities e.g. conditions such as unsuppressed HIV infection, or congenital deficiencies, or cancer malignancies; or the administration of immunosuppressant drugs such as chemotherapy for cancer or steroids.

The effect of ageing was particularly strong within our review, both in terms of the magnitude of effect estimates and the number of studies presenting evidence. As well as the above impact of comorbidities, we note that the host’s age may influence pathogenesis, both in terms of the likelihood of having various comorbidities, and also due to its effect on the immune system. Indeed, the immune system becomes less effective over time (immunosenescence), which affects the quality and number of immune system cells generated [59]. Given the scale of the impact of age documented within this review, it seems unlikely that its effect can be explained by a single or a small number of comorbidities which are yet to be detected. This opens up the need to explore biological markers, for example ACE2 [60], and markers of immunosenescence.

The strengths of our review include its systematic approach and broad use of search terms to avoid missing studies. We additionally present a quality assessment to aid the interpretation of the strength of the evidence. In some instances, included publications may have focussed on one specific outcome, whereas our quality assessment took the perspective of the outcomes extracted for this review. We were unable to detect instances where two publications used the same patient populations for their analyses, potentially over-emphasising certain findings. Given the global nature of the pandemic, our review includes studies from around the world, albeit with a large preponderance from China, including studies conducted early after the emergence of SARS-CoV-2 when the at-risk population was predominantly those who had contact with Huanan seafood market and their contacts, and not necessarily representative of the general population. We note a particular lack of studies from the African continent and the Americas, which may have implications for generalisability. Given the rapidly evolving literature on COVID-19, we also note our exclusion of studies published online after April 2020 (and the time period in which the surrounding text was written), for example the Dai report on cancer as a risk factor [61] and our exclusion of preprints (which was undertaken to ensure that all included studies had undergone an external quality assessment prior to inclusion).

Across the included publications, variability in study design, exposure and outcome measurement, and analyses made exact syntheses of effect sizes across different risk factors very difficult. Measures of disease severity varied, e.g. admission to ICUs or clinical parameters such as percentage oxygen saturation of the blood. Even measures such as admission to ICU can be subjective and may be time-, clinician-, and health systems-dependent. If severity is recorded at admission, risk factors may reflect issues associated with delayed access to healthcare, which may differ between settings and healthcare systems. It is also important to note that, in some studies of disease severity, mild disease included both people who were hospitalised with symptoms and asymptomatic individuals identified through contact tracing. Generally, analyses were descriptive or univariable and thus did not control for confounding. As documented above, this may be particularly problematic when it comes to separating the impact of age and the presence of comorbidities, as well as for identifying which comorbidities truly increase risk, given that many patients may have multi-morbidity.

The implications of our findings are two-fold for COVID-19, firstly for current public health practice and secondly for the design of future studies. We flag a number of factors of interest that should be considered by governments and public health agencies when designing shielding strategies and the targeting of future vaccines, as well as in mathematical modelling projecting the likely impact of the pandemic over time. We note, however, the need for sensitive handling of population groups deemed to be at higher risk, and how such labelling does not devolve responsibility from public bodies to these individuals for their own welfare [8]. Some public health agencies are now including reporting of potential risk factors in their routine outputs, including ICNARC (included in this review) [30] and the newer European Centre for Disease Prevention and Control reports, which were released after this review was conducted [62].

Our review demonstrates both the volume of literature that can be published within only a few months since the appearance of an emerging infectious disease, and the need for co-ordinated approaches to such pathogens. Global efforts using national datasets are hugely valuable in systematically determining the aetiology of a disease, particularly to detect smaller effect sizes. Determination of the exact threshold of important risk depends on public perceptions of the disease [63], as well as policy needs. Data collection should be standardised where possible, e.g. by using consistent definitions of outcomes and the treatment of exposures (for example for hypertension, given that blood pressure is continuous). (For COVID-19 we note both the valuable World Health Organization interim guidelines on its management in providing consistent approaches for testing and the definition of ARDS [14], and that platforms such as the International Severe Acute Respiratory and Emerging Infections Consortium (ISARIC) have aimed to facilitate such standardisation [64].) The choice of comparison groups should also merit careful consideration; comparison to other forms of the same condition (e.g. SARS and MERS for COVID-19), although interesting, provide little information about risk groups to be currently acted upon. Where key potential risk factors of interest, such as deprivation, are linked to both the disease of interest and the comparator condition, this limits the inferences possible. Saying this, studies of COVID-19 with the comparator group of other forms of viral pneumonia are a useful complement to studies using a general population comparator, as they show whether people with particular risk factors are at risk over and above what they might experience from ‘normal’ respiratory viruses, which might inform the level of additional precautions they could consider taking.

Finally, appropriately adjusted multivariable analyses should be prioritised, in order to separate the implications of different risk factors and to infer true causal relationships, for example exploring specific markers of comorbidity severity and control, such as the use of specific medications. We can then make the recommendations for shielding criteria more targeted, meaning that the public can be made more aware of the risk factors that are likely to have clinical significance and adapt their behaviour accordingly. Early clinical studies during pandemics are critically important and published rapidly under extremely difficult circumstances, but we would argue that high-quality epidemiological studies should also be seen as a priority, and that emergency response plans should include provision of appropriate epidemiological and statistical expertise.

## Conclusions

The volume of literature generated in the short time since the appearance of SARS-CoV-2 has been considerable. Many studies have sought to document the risk factors for COVID-19 disease, disease severity and mortality. Age was the only risk factor based on robust studies and with a consistent body of evidence. Mechanistic studies are required to understand why age is such an important risk factor. At the start of pandemics, large, standardised, studies using multivariable analyses – e.g. using national surveillance data – are urgently needed in order to inform stratified approaches to rapidly protecting the population groups most at risk.

## Data Availability

All data generated or analysed during this study are included in this published article and the underlying papers.

## Abbreviations

ACE2: Angiotensin-converting enzyme 2
ARDS: Acute respiratory distress syndrome
BMI: Body mass index
CDC: Center for Disease Control and Prevention
COPD: Chronic obstructive pulmonary disease
COVID-19: Coronavirus disease-19
CT: Computed tomography
DIC: Disseminated intravascular coagulation
FiO_2_: Inspired oxygen fraction
ICNARC: Intensive care national audit and research centre
ICU: Intensive care unit
IL: Interleukin
ISARIC: International severe acute respiratory and emerging infections consortium
LRT: Lower respiratory tract
MERS: Middle Eastern Respiratory Syndrome
MS: Multiple sclerosis
N/A: Not applicable
PaO_2_: Arterial partial pressure of oxygen
PCR: Polymerase chain reaction
RAS: Renin-angiotensin system
RR: Respiratory rate
SARS: Severe acute respiratory syndrome
SARS-CoV-2: Severe acute respiratory syndrome coronavirus-2
SpO_2_: Oxygen saturation
TMPRSS2: Transmembrane serine protease 2
URT: Upper respiratory tract
